# Two novel mutations in the bestrophin-1 gene and associated clinical observations in patients with best vitelliform macular dystrophy

**DOI:** 10.3892/mmr.2015.3711

**Published:** 2015-04-30

**Authors:** YING LIN, HONGBIN GAO, YUHUA LIU, XUANWEI LIANG, XIALIN LIU, ZHONGHAO WANG, WANJUN ZHANG, JIANGNA CHEN, ZHUOLING LIN, XINHUA HUANG, YIZHI LIU

**Affiliations:** 1 State Key Laboratory of Ophthalmology, Zhongshan Ophthalmic Center, Sun Yat-Sen University, Guangzhou, Guangdong 510060, P.R. China; 2 Guangdong Provincial Key Laboratory of Occupational Diseases Prevention and Treatment, Guangdong Hospital for Occupational Disease Prevention and Treatment, Guangzhou, Guangdong 510300, P.R. China

**Keywords:** best vitelliform macular dystrophy, bestrophin-1 gene, mutation

## Abstract

The purpose of the current study was to investigate the 11 *bestrophin-1* (*BEST1*) exons in patients with best vitelliform macular dystrophy (BVMD), and to characterize the associated clinical features. Complete ophthalmic examinations were conducted on two families, and two family members were diagnosed with BVMD. Genomic DNA was extracted from the leukocytes of peripheral blood collected from the patients and their family members, in addition to 100 unrelated control subjects recruited from the same population. The polymerase chain reaction was used to amplify a total of 11 exons of the *BEST1* gene, which were directly sequenced. Ophthalmic examinations, including best-corrected visual acuity, slit-lamp examination, fundus examination, fundus photography and fluorescein angiography imaging, as well as anterior segment analysis with Pentacam and optical coherence tomography, were conducted. The patients exhibited yellowish lesions in the macular area. A heterozygous mutation c.910_912delGAT (p.304del Asp) in exon 7 was identified in Case 1. A heterozygous *BEST1* missense mutation c.685T>G (p.Trp229Gly) in exon 5 was identified in Case 2, but not in any of the unaffected family members or normal controls. Although *BEST1* gene mutations and polymorphisms have previously been reported in various ethnic groups, the current study identified, for the first time to the best of our knowledge, two novel *BEST1* gene mutations in patients with BVMD.

## Introduction

Best vitelliform macular dystrophy (BVMD) is one of the most common forms of autosomal dominant macular dystrophy, characterized by the presence of yellowish lesions in the macular area (1–4).

BVMD has been classified into five phenotypic stages: The previtelliform, vitelliform, pseudohypopyon, vitelliruptive and atrophic stages. Not all patients will progress through all five stages, and the stages do not always occur consecutively. Furthermore, BVMD lesions may simultaneously display characteristics of various BVMD stages (5–8).

BVMD is associated with mutations in the *bestrophin 1* gene (*BEST1*), on chromosome 11q12, which encodes a 585 amino acid transmembrane protein and is selectively expressed in the retinal pigment epithelium (RPE) (9–11). The *BEST1* gene is the founding member of a family of four paralogs, which also includes *BEST2*, *BEST3* and *BEST4*. *BEST1* is expressed in the RPE cells of the developing and adult eye cells (1,9).

Greater than 300 distinct *BEST1* mutations have been identified in families or sporadic patients affected by BVMD (12–17). It has been hypothesized that mutations in *BEST1* may result in channel dysfunction which leads to abnormal fluid and ion transport through the RPE, resulting in defects during ocular growth and later-onset retinal dystrophy (18,19).

Mutations in the *BEST1* gene are detected in the majority of cases of BVMD with a positive family history. The current study aimed to conduct mutational analysis of two Chinese families with BVMD at the gene level, in addition to analyzing the associated clinical features.

## Patients and methods

### Ethical approval

Approval for the current study was provided by the ethics committee of Zhongshan Ophthalmic Center, Sun Yat-Sen University (Guangzhou, China). Two patients were diagnosed with BVMD at the Zhongshan Ophthalmic Center.

### Ophthalmic examinations

A variety of techniques were used for the ophthalmic examinations, which are outlined as follows: Visual acuity was examined using the Early Treatment Diabetic Retinopathy Study chart (Precision Vision, LaSalle, IL, USA). Anterior segment photographs were captured using a BX 900 Slit Lamp (Haag-Streit AG, Köniz, Switzerland). Anterior segment measurements were taken with Pentacam^®^ HR version 70700 (OCULUS Optikgeräte GmbH, Wetzlar, Germany). Fundus photography and fundus fluorescein angiography (FFA) imaging was performed using a Heidelberg Retina Angiograph (Heidelberg Engineering GmbG, Heidelberg, Germany). In addition, physical examinations, including blood examination, a urine test, electrocardiogram, chest X-ray, blood biochemistry test, blood lipid and blood coagulation tests were conducted to exclude systemic diseases.

### Sample collection

Two affected families were identified as described. A total of 100 subjects who exhibited no diagnostic features of BVMD were recruited from the same population to serve as normal controls. Written informed consent was obtained from all participating individuals prior to the commencement of the current study. According to the principles of the Declaration of Helsinki, venous blood samples were collected from peripheral blood leukocytes for genomic DNA extraction using a DNA extraction kit (Qiagen, Hilden, Germany).

### Mutation detection

Exons of the *BEST1* gene were amplified with the polymerase chain reaction (PCR), using the primers (Beijing Genomics Institute, Guangzhou, China) presented in Table I (20). In brief, PCR was performed in a 50-*µ*l total volume reaction. All reagents used for PCR were purchased from (Takara Bio, Inc., Tokyo, Japan). The PCR cycling profile was as follows: One cycle at 94°C for 5 min, followed by 40 cycles at 94°C for 45 sec, 52–66°C for 45 sec and 72°C for 45 sec, in addition to one cycle at 72°C for 10 min. The PCR products were sequenced from both directions using an ABI3730 Automated Sequencer (Applied Biosystems Life Technologies, Foster City, CA, USA). The sequencing results were analyzed using SeqMan, version 2.3 (Technelysium Pty Ltd., Brisbane, Australia) and they were compared with the reference sequences in the database at the National Center for Biotechnology Information (NC_000011.9; http://www.ncbi.nlm.nih.gov/nuccore/224589802).

## Results

### Clinical data

**Figure 1:**
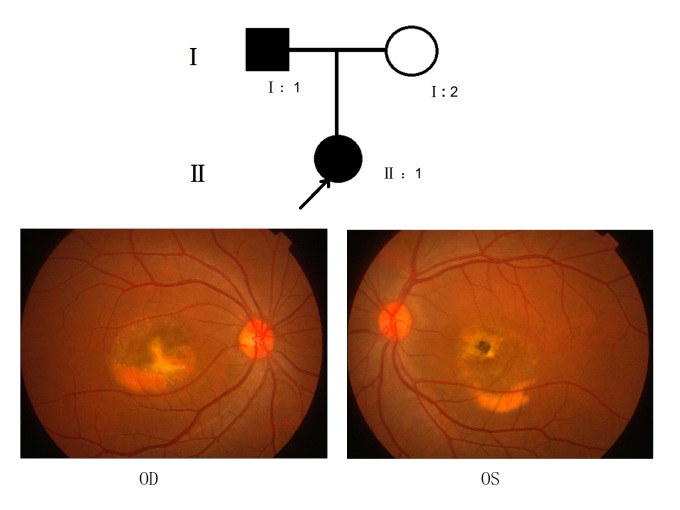
Pedigree of Family 1. Square symbols denote males, and circular symbols denote females. The shaded symbols indicate ophthalmologist-confirmed best vitelliform macular dystrophy. The arrow indicates the proband (II:1). Fundus examination revealed vitelliruptive lesions with a scrambled egg-like appearance and dispersion of the vitelliform material, with signs of atrophy in the left eye of Case 1 (II:1) (magnification, ×50). OD, oculus dexter; OS, oculus sinister.

**Figure 2:**
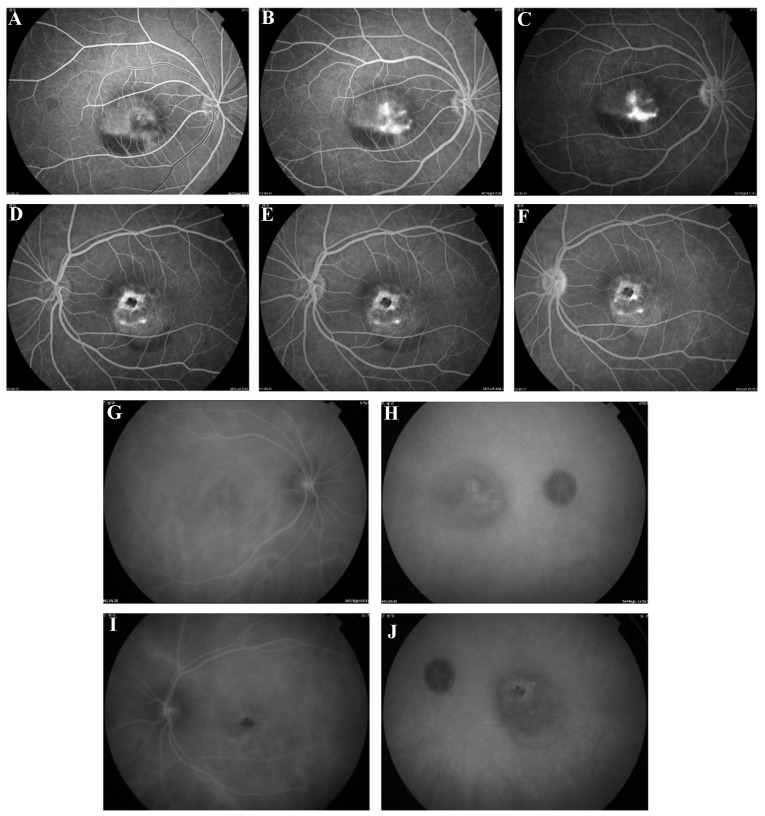
Fundus photography, FFA imaging and ICG were performed using a retina angiograph. (A) FFA revealed significant early hyperfluorescence (B and C) that increased in intensity at the late stage of the angiographic sequence (D–F) and was associated with moderate leakage in the right eye and macular lesions that simulated a pattern of dystrophy in the left eye. (G and I) ICG detected hypofluorescence in the early stages of the angiographic sequence in the left and right eyes and subsequently detected (H and J) little abnormal hyperfluorescence at the late stages (magnification, ×50). ICG, indocyanine green chorioangiography; FFA, fundus fluorescein angiography.

The patients evaluated in the current study were from the southern area of China. Case 1 (II:1) of Family 1 was a 23-year old female with a family history (Fig. 1) of ocular disease, diagnosed with BVMD at the age of 21 years. Her best-corrected visual acuity, as measured by the Logarithm of the Minimum Angle of Resolution (LogMAR) using the following formula: LogMAR visual acuity = 0.1 + LogMAR value of the best line read −0.02 X (number of letters read), was 0.1 in the right eye and 0.4 in the left eye. Examination of the fundus identified vitelliruptive lesions with a scrambled egg-like appearance and dispersion of the vitelliform material, including signs of atrophy, in the left eye (Fig. 1; OS). Fluorescein angiography (Fig. 2) demonstrated significant early hyperfluorescence (Fig. 2A) that increased in intensity at the late stage of the angiographic sequence, and was associated with moderate leakage (Fig. 2B and C) in the right eye, and macular lesions that simulated a pattern of dystrophy in the left eye (Fig. 2D–F). Indocyanine green chorioangiography (ICG; Fig. 2G–J) detected hypofluorescence in the early stages (Fig. 2G and I) of the angiographic sequence in the left and right eyes and subsequently detected little abnormal hyperfluorescence the late stages (Fig. 2H and J). The father of Case 1 was also diagnosed with ocular disease during his teenage years, and at the time of the present study, exhibited marked macular degeneration and cataracts.

**Figure 3:**
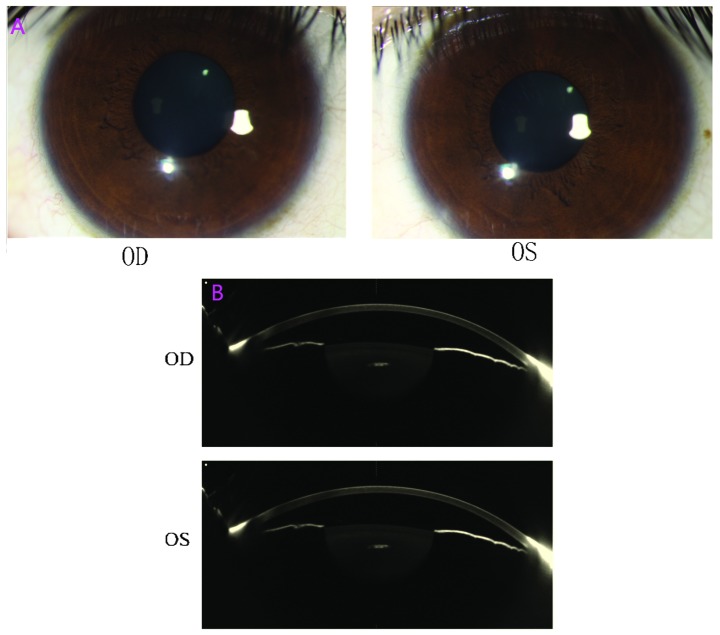
(A) Anterior segment photograph of Case 1 captured with a BX 900 Slit Lamp. (B) Anterior segment photograph of Case 1 captured with a Pentacam. The anterior chamber depths were 2.09 mm (OD) and 2.12 mm (OS), and the central corneal thickness was demonstrated to be 548 *µ*m (OD) and 558 *µ*m (OS) (magnification, ×50). OD, oculus dexter; OS, oculus sinister.

The anterior segment photograph captured using a BX 900 Slit Lamp is presented in Fig. 3A, and the images presented in Fig. 3B were captured using Pentacam. The anterior chamber depths were 2.09 mm [oculus dexter (OD)] and 2.12 mm [oculus sinister (OS)], and the central corneal thickness was identified to be 548 *µ*m (OD) and 558 *µ*m (OS).

**Figure 4:**
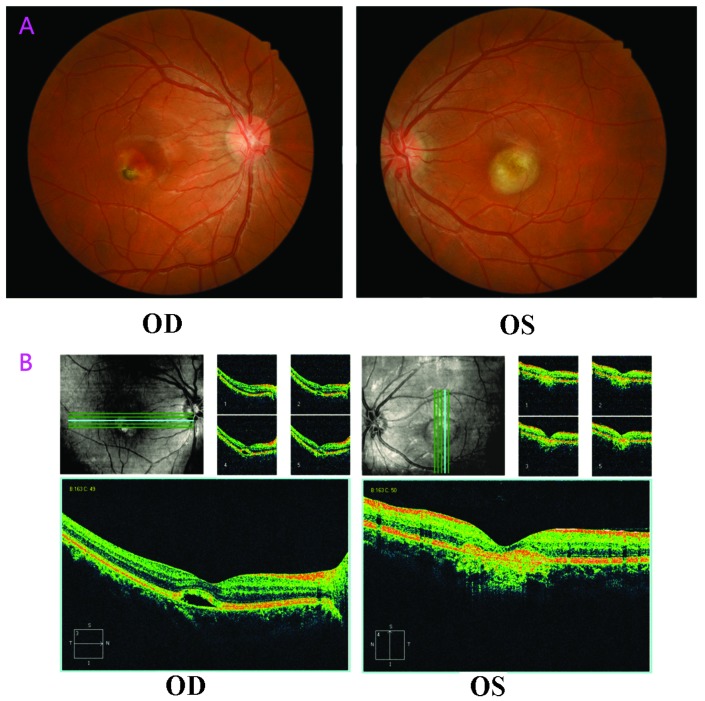
(A) Examination of the fundus of Case 2. Vitelliruptive lesions with a scrambled egg-like appearance were observed, in addition to dispersion of the vitelliform material with signs of atrophy in the right eye. (B) Spectral domain optical coherence tomography scans demonstrated clear abnormalities, including the absence of the foveal pit, serous retinal detachment, cystoid macular edema and interruption of the outer limiting membrane. The right eye exhibited marked yellow-white subretinal scarring with pigmented borders, surrounded by serous retinal detachment. In addition, the left eye exhibited an atrophic lesion (magnification, ×50). OD, oculus dexter; OS, oculus sinister.

Case 2 of Family 2 was a 22 year-old male with no known familial history of ocular disease at the time of diagnosis of BVMD aged 19 years. His best-corrected visual acuity, as measured by LogMAR, was 0.7 in the right eye and 0.4 in the left eye. Fundus examination (Fig. 4A) indicated vitelliruptive lesions with a scrambled egg-like appearance and dispersion of the vitelliform material with signs of atrophy of the right eye. This patient was identified to be supersensitive to fluorescein, therefore no FFA or ICGA results were obtained.

The anterior chamber depths were 3.15 mm (OD) and 3.17 mm (OS), and the central corneal thickness was identified to be 564 *µ*m (OD) and 568 *µ*m (OS). Spectral domain optical coherence tomography (OCT) scans revealed clear abnormalities, including the absence of the foveal pit, serous retinal detachment, cystoid macular edema and interruption of the outer limiting membrane (Fig. 4B). The right eye was observed to exhibit prominent yellow-white subretinal scarring with pigmented borders, surrounded by a serous retinal detachment. Additionally, the left eye presented with an atrophic lesion.

OCT revealed that the foveal region of the right eye was unusually thick due to the presence of abnormal neuroretinal detachment from the RPE, which was suggested to have been triggered by an aberrant accumulation of fluid within the choriocapillaris and between the RPE and the fovea. In addition, OCT indicated that the foveal region of the left eye was abnormally thick due to irregularities in the deep retinal layers, with aberrant junctions between the inner and outer segents, as well as a prominent and hyperreflective structure in contact with the overlying retina, which was surrounded by elevated retina with underlying spots of increased reflectivity.

### Mutation screening

**Figure 5:**
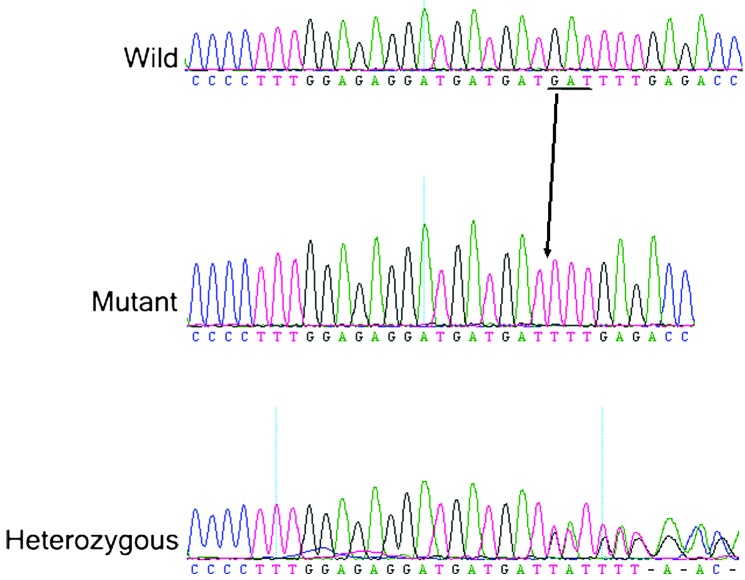
A heterozygous mutation c.910_912delGAT (p.304del Asp) in exon 7 of *BEST1* was identified in the affected members of Family 1.

**Figure 6:**
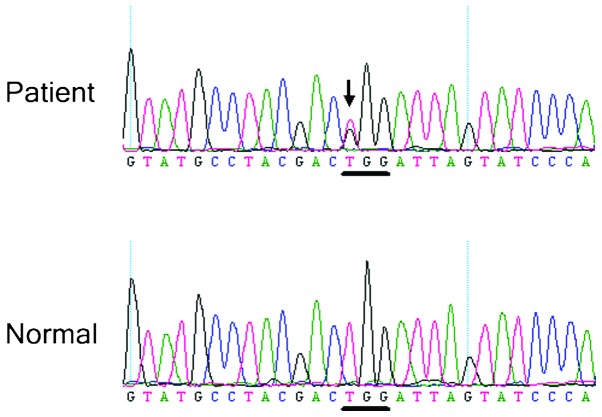
A heterozygous *BEST1* missense mutation c.685T>G (p.Trp229Gly) in exon 5 was identified in the affected Case 2 (Family 2).

A heterozygous mutation c.910_912delGAT (p.304del Asp) was identified in exon 7 in the affected family members of Family 1 (Fig. 5). A heterozygous *BEST1* missense mutation c.685T>G (p.Trp229Gly) in exon 5 was identified in the affected Case 2 (Fig. 6), but not the unaffected members of Family 2 or the normal controls. These identified mutations had not previously been reported.

## Discussion

BVMD is associated with mutations in the *BEST1* gene, which was formerly known as the *VMD2* gene. The *BEST1* gene encodes bestrophin-1, a transmembrane protein located in the basolateral membrane of the RPE (21–23).

A large number of allelic variants of the *BEST1* gene have previously been identified and are recorded in the Regensburg University (Bavaria, Germany) database (24).

In the current study, two mutations were identified in the *BEST-1* gene, which are associated with Best Syndrome: c.910_912delGAT (p.304del Asp) and c.685T>G (p.Trp229Gly). These mutations, rather than a rare polymorphism in the normal population, are suggested to be the causative mutations in the two sporadic BVMD patients.

Although >300 distinct *BEST1* allelic variants have been identified in BVMD, a comprehensive database summarizing the known associations between *BEST1* mutations and the prognosis of patients with BVMD worldwide remains to be developed. This is due to the fact that systematic whole *BEST1* gene sequencing and clinical examinations have not been completed in a sufficiently large cohort of affected patients.

BVMD is a bilateral, symmetric, progressive disease of the macular area, associated with long-term loss of vision. Onset of the disease is predominantly early in life, often occurring by 10 years of age (7,25,26). Amongst certain patients, BVMD rapidly progresses towards the loss of central vision (22,26); thus, a thorough investigation of the age of onset of this disease may aid the development of valuable diagnostic/prognostic techniques. Conducting studies on independent cohorts of children belonging to families with a known history of BVMD may also be beneficial.

In conclusion, the current study identified two novel mutations of the *BEST1* gene in two Chinese families with BVMD. The results of the current study expanded the library of known mutations of *BEST1* and are valuable for the development of genetic counseling and prenatal diagnosis in families with BVMD.

## Figures and Tables

**Table I:** Primers used for the polymerase chain reaction.

Exon	Forward (5′-3′)	Reverse (5′-3′)	Product size (bp)	Annealing temperature (°C)
2	AGTCTCAGCCATCTCCTCGC	TGGCCTGTCTGGAGCCTG	212	61
3	GGGACAGTCTCAGCC ATCTC	CAGCTCCTCGTGATCCTCC	238	58
4	AGAAAGCTGGAGGAGCCG	GCGGCAGCCCTGTCTGTAC	1408	59
5	GGGGCAGGTGGTGTTCAGA	GGCAGCCTCACCAGCCTAG	150	59
6	GGGCAGGTGGTGTTCAGA	CCTTGGTCCTTCTAGCCTCAG	181	59
7	CATCCTGATTTCAGGGTTCC	CTCTGGCCATGCCTCCAG	257	59
8	AGCTGAGGTTTAAAGGGGGA	TCTCTTTGGGTCCACTTTGG	215	59
9	ACATACAAGGTCCTGCCTGG	GCATTAACTAGTGCTATTCTAAGTTCC	298	59
10A	GGTGTTGGTCCTTTGTCCAC	CTCTGGCATATCCGTCAGGT	591	59
10B	CTTCAAGTCTGCCCCACTGT	TAGGCTCAGAGCAAGGGAAG	457	59
11	CATTTTGGTATTTGAAATGAAGG	CCATTTGATTCAGGCTGTTG	216	59

Summary of the primers and product lengths used for the amplification of the 11 exons of *BEST1*. bp, base pairs.
